# A Decade of the GSTT Aortic Infection Programme: A Description of a Clinical Management Algorithm by a Multidisciplinary Team

**DOI:** 10.3390/jcm15051754

**Published:** 2026-02-25

**Authors:** Thomas Richards, Carolyn Hemsley, Nicholas Price, Morad Sallam

**Affiliations:** Guy’s and St Thomas’ NHS Foundation Trust, London SE1 9RT, UK

**Keywords:** aortic aneurysm, aneurysm, infected, patient care team

## Abstract

**Background/Objectives**: The management of mycotic aortic aneurysm and aortic graft infection is complex, and the pathology is rare. Over the past decade, we have developed a high-volume multidisciplinary service and comprehensive management algorithm. We intend to report on our experience and management strategy. **Methods**: We have examined the different aspects of the service, how it was developed, the standardisation of management, and its impact on consistent decision-making. **Discussion**: Low-volume, high-complexity pathologies are challenging and infrequently reported in the literature. The growth of our service and experience has improved outcomes and allowed for timely and consistent decision-making. We believe a central, multidisciplinary, standardised approach allows for prospective data collection and analysis. Although mycotic aortic aneurysm and aortic graft infection are discrete pathologies, our management algorithm and dedicated aortic infection service provides a comprehensive management strategy for both conditions. **Conclusions**: A comprehensive management algorithm has not previously been described by a tertiary centre. We have demonstrated that a unique multidisciplinary clinical approach and dedicated specialist service following a standardised management algorithm delivers superior patient outcomes. Whilst our basic management algorithm has remained largely intact over the years, we anticipate change in the future based upon emerging high-quality evidence and our own experience.

## 1. Introduction

Aortic graft infection (AGI) is a serious complication of both open and endovascular aortic surgery with high mortality rates and significant morbidity. The pathology is both rare and complex, with an incidence between 1 and 2% of patients receiving aortic grafts [1]; the incidence is similar for open surgical repair (OSR) and endovascular aneurysm repair (EVAR).

Following haematological seeding or contiguous spread of infection from surrounding structures, the natural progression of untreated AGI has protean clinical manifestations ranging from pain and constitutional symptoms (e.g., fever, weight loss and fatigue) to life-threatening bleeding, fistulation and severe sepsis. Infection of the synthetic graft material used for aortic reconstruction is typically difficult to treat conservatively and a fundamental tenet of successful management is surgical ‘source control’ by explantation of all infected material. Mortality for patients with AGI who do not undergo explantation surgery has been reported to be 70–100% at two years, despite antibiotic therapy [2,3].

Depending on the nature and site of the infected endograft, however, complete surgical explantation is alassociated with significant mortality [2]. Strategies include either complete or partial removal of the infected synthetic material, and reperfusion of the viscera, pelvis and lower limbs is achieved by either in situ reconstruction or extra-anatomical bypass of the aorta. In situ reconstruction requires a biological conduit, for which an autologous vein, cadaveric human aorta and bovine pericardium are options, each with specific nuances to their use.

Low-volume, high-complexity surgery generates variability in practice both between and within units. Superiority of a particular technique is difficult to recognise and subsequently report, which is reflected by a lack of evidence in the literature. This provides an opportunity for inferior practices to persist.

Due to variability in the complexity and severity of pathology, and the low incidence and volume of interventions, the mortality of patients treated surgically for AGI is difficult to interpret accurately from the literature; however, we have previously found the overall 30-day mortality to be 30% [2]. Comparison of different treatment modalities is particularly challenging; a 2024 systematic review and meta-analysis found mortality to be 6.1–41.4% at one year and 32.1–90% at three years, depending on the surgical strategy [4]. This is acknowledged in the European Society for Vascular Surgery’s (ESVS’s) 2020 guidelines for the management of aortic graft and endograft infections, which summarises the relevant evidence and makes general recommendations [5].

We believe our algorithm provides more specific recommendations than the ESVS’s guidelines, most notably the cessation of antimicrobials, active primary endovascular management of mycotic aneurysms and integration of PET-CT as an adjunct [5].Our experience also suggests that the development of management algorithms and subsequent evaluation of programme outcomes are more useful than comparing specific operations.

Our unit’s observation of the poor outcomes associated with conservative managed AGI [2] prompted a turning point in our practice and resulted in formation of a multidisciplinary team (MDT) working dedicated to the management of AGI. Since 2016, we have seen our aortic infection practice, particularly regarding the management of AGI, grow from an ad hoc service for complications of aortic surgery within our catchment to a national referral service with the highest volume in the country.

We reported our medium-term outcomes of patients undergoing treatment for AGI between 2015 and 2020 in 2023 [6]. A total of 54 patients were referred to the service, of which 45 (83%) underwent complete explantation of an infected aortic graft and in situ biological reconstruction. The overall survival rate was 73% at a median follow-up of two years. There were no perioperative deaths, and two patients died within 30 days of surgery [6].

Our aim here is to describe the development and implementation of a standardised MDT-based AGI management algorithm and propose a reproducible model for other tertiary centres to consider adopting. Our medium- to long-term outcomes are awaiting publication separately.

Throughout the description of our service, we make recommendations in a field where there is a paucity of high-quality evidence, which is inevitably is based upon our growing institutional experience and expert consensus.

## 2. GSTT Aortic Infection Service

From the outset, we intended to establish a dedicated multidisciplinary service with a standardised approach to all patients referred with suspected or confirmed infections of the native aorta or AGI.

### 2.1. Aortic Infection MDT

We hold a weekly MDT which is run by a dedicated senior nurse MDT coordinator. The MDT attendees are consultants in Infectious Diseases and Vascular Surgery and Clinical Nurse Specialists (CNSs) in Vascular Surgery and Outpatient Parenteral Antibiotic Therapy (OPAT).

Consultants in Vascular Anaesthesia, Nuclear Medicine, Interventional Radiology and Perioperative care for Older People undergoing Surgery (POPS) contribute to decision-making outside of the MDT on an as-required basis. Surgeons from other surgical specialties also contribute on a case-by case basis where surgical strategy would require their input, for example due to fistulation, the requirement of cardiopulmonary bypass or spinal osteomyelitis.

Regional, national and international referrals of patients with AGI are initially made to the MDT coordinator by email or letter. Referrers are provided with a proforma to complete, and once all required information plus relevant imaging has been compiled, the patient is added to the next MDT. Referring surgeons and physicians are invited to attend the MDT for their patient if they express an interest in doing so.

### 2.2. Standardised Diagnosis

**Figure 1:**
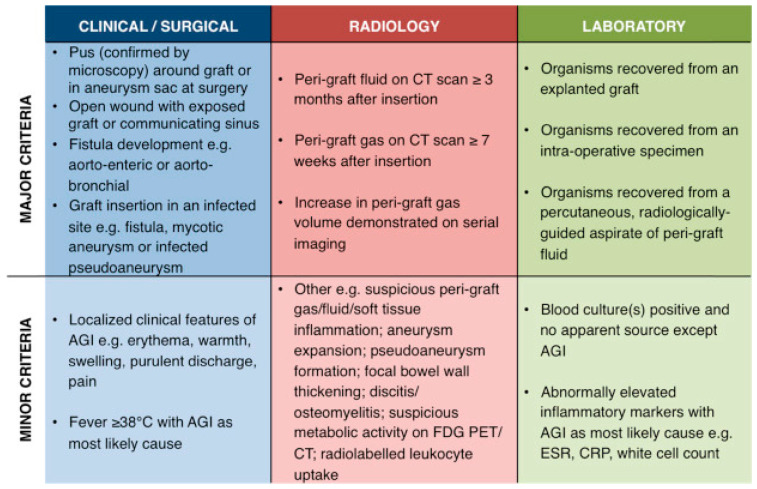
MAGIC criteria.

Diagnosis for AGI is made according to the Management of Aortic Graft Infection Collaboration (MAGIC) [7] criteria, which originated from our centre. This describes major and minor signs of AGI separated into three categories of clinical, radiological and laboratory signs. It states that AGI should be suspected if a single major criterion or two or more minor criteria from different categories are present, and AGI is diagnosed if there is any one major criterion and any criterion from another category (Figure 1).

As experience has grown, we have incorporated Positron Emission Tomography–Computed Tomography (PET-CT) into our diagnostic algorithm, particularly since we recognise its clinical value and have reliable timely access to PET-CT on our site. In consultation with the Nuclear Medicine consultants, we have standardised how AGI investigations are reported and we use PET-CT as an adjunct to MAGIC criteria in difficult-to-diagnose cases, surgical planning, and follow-up progression and disease control as a surveillance and prognostic imaging tool.

Outcomes from the MDT include recommendations for the selection, dose and duration of antimicrobial therapy, further investigations if indicated and specialist consultations for counselling regarding the different management options.

In the 10 years since the MDT was implemented, more than 270 patients with suspected or confirmed diagnosis of native aortic infection or AGI have been discussed, of which 127 were confirmed to have AGI, and 101 patients have undergone explantation of the infected graft and aortic reconstruction. We currently receive approximately 20–30 new referrals a year, of which two-thirds undergo surgery.

### 2.3. Aortic Infection Clinic

The Aortic Infection Clinic runs for 3–4 h once a month and is attended by consultants in Infectious Diseases and Vascular Surgery and a Vascular Surgery CNS.

For patients who have travelled long distances, we offer a one-stop assessment, work-up and consultation, with accommodation on-site to enable PET-CT, any further investigation, and review by the POPS team to be undertaken on the same visit as the outpatient appointment.

### 2.4. GSTT Aortic Infection Algorithm

To our knowledge, a management algorithm to guide the approach to diagnosis and different treatment options of both mycotic aortic aneurysms and AGI has not been published. Over the last 10 years, our dedicated aortic infection programme has developed an algorithm to standardise practice and guide decision-making, also enabling us to record prospective data to further guide our decisions of treatment strategies.

Our collective mindset has evolved from attempting to establish the best treatment option to accepting that each option is suitable for a specific patient; our algorithm allows us to select the best treatment option for a particular patient in a timely fashion.

**Figure 2:**
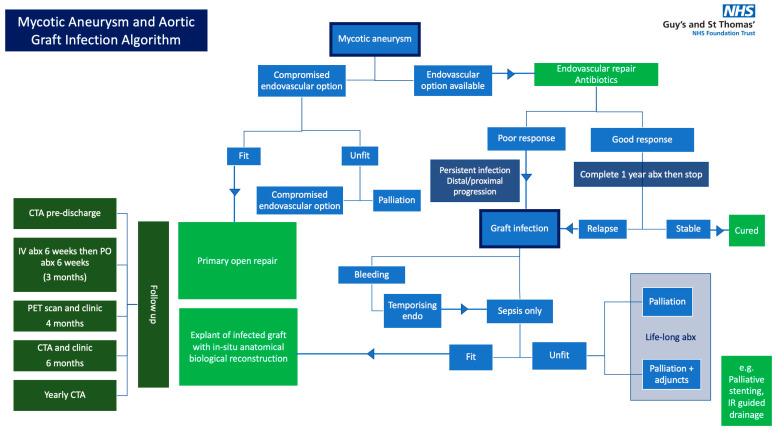
Aortic infection algorithm.

The algorithm incorporates the management of both mycotic aneurysms of the native aorta and AGI; these are different pathologies, although there is overlap between their pathophysiology and management (Figure 2).

We specify that patients with untreated mycotic aortic aneurysm or AGI who are bleeding from any aortic fistula should be treated endovascularly as an emergency, before re-entering the algorithm as a potential AGI.

Key decision points are:

1. Assessment of suitability for endovascular treatment of mycotic aneurysm: if endovascular treatment is thought to be compromised, a fit patient would be considered for primary open repair and an unfit patient would be considered for compromised endovascular treatment or palliation.
2. Failure of conservative management: if response to antimicrobial therapy is insufficient or an infection recurs.
3. Fitness for explantation: this is considered on a case-by-case basis in consultation with Anaesthetics and POPS before MDT discussion.

### 2.5. Mycotic Aortic Aneurysms

Using the MAGIC criteria, an endograft inserted into an infected field is automatically regarded as a *de facto* graft infection and over time, we have come to consider ‘stented’ mycotic aneurysms as a distinct subset of graft infection that may be managed medically.

Current evidence [8] reports that up to a third of patients with mycotic aneurysms of their native aorta can be definitively treated with EVAR and antibiotic therapy without needing subsequent device removal by open surgery. Multiple factors are likely to contribute to the success of conservative treatment, including administration of effective antimicrobials for a period *before* stent insertion, positive microbiology enabling targeted treatment, and pathogen-specific properties that increase adhesion to prosthetic surfaces and reduce antimicrobial effectiveness, e.g., biofilm production by *Staph. aureus*.

Cessation of antimicrobial therapy is considered possible if inflammatory indices have normalised AND suspicious evidence of ongoing infection is absent on PET-CT scanning. The duration of antibiotic treatment depends on the specific circumstances and ranges between 12 weeks and 12 months. If conservative management fails, device removal and open repair is indicated. If explantation is felt to be associated with an unacceptable risk, then long-term antimicrobial suppression is an option, which may palliate symptoms but is not expected to prolong life. Of note, we do not expect it is possible to cure infections caused by *Staph. aureus* conservatively.

Experience has therefore led us to favour an ‘endo-first’ approach, with structured opportunities to test the continuing response to antimicrobial therapy and its subsequent cessation. We consider this testing of a less invasive treatment to be the best mitigation of the risks of unnecessary highly morbid open surgery and the drawbacks of life-long antibiotics.

### 2.6. Standardised Surgical Management Principles

Open surgical management is necessary for the following, considered to represent established aortic graft infection and requiring ‘source control’ to effect complete cure:

1. Failure of conservative management for stented mycotic aneurysms (above) and/or where ‘bridging’ endografts have been deliberately deployed as a temporising measure.
2. Aortic graft infection involving a pre-existing, non-biological prosthesis, e.g., open repair with PTFE performed several years ago.
3. Any situation with fistulation present, e.g., aorto-enteric fistula.

Pre-operative management is standardised and includes comprehensive assessment and optimisation by a Vascular Anaesthetist and the POPS team, with some patients undergoing prehabilitation including pre-operative nutritional support.

Our standard surgical strategy involves complete removal of the infected graft, extensive local debridement and in situ reconstruction with biological material (bovine pericardium, autologous vein or a composite according to anatomical needs and tissue availability). The choice is tailored to the specific need of the patient.

Careful attention is paid to collecting intraoperative specimens for microbiology. As outlined in the MAGIC protocol, five specimens are collected using separate instruments and sample containers to maximise diagnostic yield and assist with interpreting possible contaminant organisms.

Surgery is undertaken by a dedicated team with a lead vascular surgeon; a cardiac surgeon and their perfusion team when indicated for thoracic aortic pathology; and oesophagogastric, colorectal, urological and spinal surgeons as required. Anaesthesia is provided by one of two cardiovascular anaesthetists experienced in anaesthesia for complex aortic surgery.

Comparative outcome analysis of surgical technique and conduit type is beyond the scope of this manuscript.

### 2.7. Standardised Medical Management Principles

Pre- and post-operative antimicrobials are directed by the Infectious Diseases team. Historically, the duration of treatment (counted from the date of surgical intervention) is 12 weeks in total, performed intravenously for the first six weeks, followed by a further six weeks parenterally if there is a suitable oral ‘switch’ agent. Table 1 shows the initial empiric antimicrobial choice depending on the clinical situation. In contrast to some older published recommendations, we do not consider the identity of the underlying pathogen to relate helpfully to the time elapsed after surgical graft implantation. Of note, Candida is a normal commensal of the human gastrointestinal tract and we have learnt to routinely include an anti-Candida agent for all patients with evidence of aorto-enteric fistula, irrespective of negative fungal culture results.

Once microbiological results are back, therapy is targeted at specific organisms that have been isolated from cultures, e.g., from blood, CT-guided aspirates, and intraoperative cultures. Molecular tests (i.e., polymerase chain reaction (PCR) assay) are requested if infection is suspected but standard microbiology cultures are negative.

Our experience shows that the range of possible pathogens is extremely wide. For ‘stented’ mycotic aortic aneurysms, the most common single causative organisms are *Staph. aureus* and *Salmonella* species. In our cohort, *Staphylococci* (*Staph. aureus* and coagulase-negative *Staph.*) form the largest group overall and polymicrobial infections, especially involving *Candida* and Enterobacteriaceae, are highly suggestive of an aorto-enteric fistula. Contrasting with practice in some other prosthetic infection settings, we do not routinely extend antibiotic treatment duration for infections involving MRSA or *Pseudomonas*.

The Infectious Diseases OPAT service is essential and wherever possible, patients benefit from early discharge and IV antibiotic therapy at home once judged medically fit for discharge. The Infectious Diseases team are responsible for directing the choice and monitoring tolerability, safety and efficacy of antimicrobial therapy.

**Figure 3:**
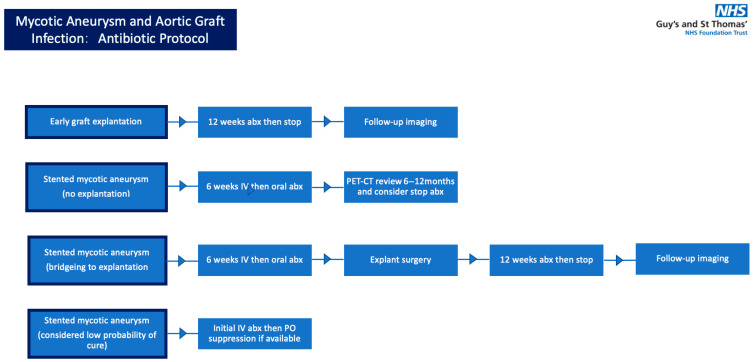
Antibiotic protocol.

Following on from IV therapy, selection of oral antimicrobial treatment is based on a combination of pathogen susceptibility, bioavailability, side-effect profile and tolerability. In a small minority of cases, all treatment may be administered intravenously if the oral ‘switch’ is too complex or if there is no oral alternative based on laboratory results. If long-term suppression rather than cure is the aim, simplicity, tolerability and low risk of resistance are prioritised (e.g., tetracyclines and co-trimoxazole). In addition, where possible, a single agent is used, sometimes at a lower than standard dose and/or frequency under expert supervision. Rather than considering suppression as a life-long commitment, we routinely reassess after 12 and 24 months, with discontinuation being a reasonable option for some (Figure 3).

### 2.8. Standardised Follow-Up Protocol

Post-operative follow-up involves preforming a CT angiogram before hospital discharge, PET-CT and clinic review at four months, and CT angiogram and clinic review at six months. Patients are surveilled with yearly CT angiograms which can be undertaken locally with the images sent to our MDT for expert review. After five years of satisfactory CT surveillance, exclusive abdominal aortic pathology can be monitored using ultrasound.

Inflammatory indices are performed whenever imaging is undertaken as a minimum. Normal inflammatory indices are good evidence that active infection is absent or under good control but must be interpreted with caution whilst antimicrobial therapy is being administered. Normal indices from 12 weeks after cessation of antimicrobial treatment are considered very likely to herald a curative outcome post-surgery.

Patients who do not undergo surgery are managed with formal palliative care or conservative best supportive care including the addition of long-term antimicrobials. Adjuncts including stenting and radiologically guided drainage are also considered. Patients referred to Palliative Care are offered counselling.

Costa et al.’s 2023 ‘Infection of Vascular Prostheses: A Comprehensive Review’ [9] provides a contemporary overview of all aspects of vascular graft infections, including aortic graft infections. It reports similar perspectives to our experience, including the predominance of staphylococcal pathogens, the role of advance imaging in diagnosis, and the need for individualised surgical and microbial strategies.

## 3. Conclusions

As with most high-complexity, low-volume pathologies, patients with infection of their native aorta or AGI remain challenging to manage. We have demonstrated that a unique multidisciplinary clinical approach, following a standardised management algorithm, delivers superior patient outcomes [6]. We recognise that we are fortunate to have a well-resourced infrastructure supporting our relatively large AGI service and advise caution in attempting to reproduce our model at a lower-volume centre.

Since high-quality research is scarce in this area, significant variation in practice and associated outcomes, even amongst tertiary referral centres, exists. In the absence of evidence, our approach over the last decade has therefore necessarily involved significant expert multidisciplinary opinions in decision-making. Whilst our basic management algorithm has remained largely intact over the years, we anticipate change in the future based upon advances in interpreting PET-CT imaging in this context. It may also be possible to reduce the duration of antibiotic treatment in specific clinical circumstances.

With multiple surgical options available and a low volume of cases, we advocate for referral to specialist centres operating a multidisciplinary model of care and hope that our structured MDT-based algorithm might inspire a similar approach in other units. Finally, comparing outcomes using the standardised algorithms employed by tertiary services using a research framework for evaluating complex interventions [10] could be more fruitful than traditional randomised controlled trials focussing on specific treatment options.

## Figures and Tables

**Table 1:** Empiric intravenous antimicrobial treatment for AGI.

EMPIRIC INTRAVENOUS ANTIMICROBIAL TREATMENT FOR AORTIC GRAFT INFECTION		
**Pre-existing non-biological graft (no fistula)**	piperacillin–tazobactam + vancomycin	
**Graft with aorto–enteric fistula**	ceftriaxone + metronidazole (+fluconazole if confirmed fistula)	
**Stented mycotic aneurysm (no prosthetic material before index endograft insertion)**	ceftriaxone	
**Additional considerations**	*Severe beta-lactam allergy*	vancomycin + ciprofloxacin (+metronidazole if possible aorto-enteric fistula)
	*Severe sepsis/haemodynamically unstable*	Add stat gentamicin pending urgent intervention
	*MRSA colonised*	Add or substitute vancomycin

## Data Availability

We have not generated or reported research data.

## Abbreviations

The following abbreviations are used in this manuscript:

AGI	Aortic Graft Infection
EVAR	Endovascular Aneurysm Repair
OSR	Open Surgical Repair
MDT	Multidisciplinary Team
CNS	Clinical Nurse Specialist
OPAT	Outpatient Parenteral Antibiotic Therapy
POPS	Perioperative Care for Older People Undergoing Surgery
MAGIC	Management of Aortic Graft Infection Collaboration
CT	Computed Tomography
PET-CT	Positron Emission Tomography–Computed Tomography
PCR	Polymerase Chain Reaction
HCLV	High-Complexity Low-Volume (Surgery)
